# High CRP-albumin ratio is associated high thrombus burden in patients with newly diagnosed STEMI

**DOI:** 10.1097/MD.0000000000035363

**Published:** 2023-10-13

**Authors:** Mustafa Kaplangoray, Kenan Toprak, Ramazan Aslan, Edhem Deveci, Ahmet Gunes, İsa Ardahanli

**Affiliations:** a Department of Cardiology, Bilecik Şeyh Edebali University, Bilecik, Turkey; b Department of Cardiology, Harran University Faculty of Medicine, Sanliurfa, Turkey; c Department of Cardiology, University of Health Sciences Mehmet Akif İnan Research and Training Hospital, Sanliurfa, Turkey.

**Keywords:** C-reactive protein-albumin ratio, primary percutaneous coronary intervention, ST-segment elevation myocardial infarction, thrombus burden

## Abstract

In patients undergoing primary percutaneous coronary intervention (pPCI) due to ST-segment elevation myocardial infarction (STEMI), an increased intracoronary thrombus burden is a strong predictive factor for adverse cardiovascular events. The C-reactive protein (CRP)-serum albumin (SA) ratio (CAR), used as an inflammatory marker, is closely associated with thrombogenicity. In this study, we investigated the relationship between coronary thrombus burden and CAR in patients undergoing pPCI due to newly diagnosed STEMI. A total of 216 patients who underwent pPCI due to STEMI were retrospectively included for the study. Angiographic thrombus burden was assessed according to thrombolysis in myocardial infarction (TIMI) grading, and those with grade 1, 2, 3 were classified as low thrombus burden (n = 120) and those with grade 4, 5 were classified as high thrombus burden (HTB) (n = 96). CAR was calculated as the ratio of CRP to SA. The average age of the patients was 60 ± 9.8, and the male ratio was 61.1. Compared to the LTB group, the HTB group had higher CAR, age, SYNTAX score, baseline cTnT, peak cTnT, CRP, glucose, WBC, and NLR while the LVEF and SA levels were lower (*P* < .05). Spearman’s correlation analysis revealed a significant correlation between thrombus burden and CAR. The multivariable logistic regression analysis revealed that CAR (odds ratio: 10.206; 95% confidence interval: 2.987–34.872, *P* < .001) was a independent risk factor for HTB. According to the receiver operating characteristic (ROC) analysis, when the cutoff value for CAR was taken as ≥1.105 CAR could predict HTB with a sensitivity of 70.8% and specificity of 67.7%. Our data indicate that CAR an independent risk factor for thrombus burden.

## 1. Introduction

Occlusive thrombus formation forms the basis of the pathogenesis of ST-segment elevation myocardial infarction (STEMI).^[1]^ After plaque rupture, thrombogenic factors that mix into the blood cause thrombus formation, leading to platelet activation.^[2]^ In STEMI, the rapid and effective restoration of blood flow in the artery associated with the infarct is the most significant factor affecting myocardial damage, ventricular functions, and long-term mortality. In contrast, thrombus burden in patients undergoing primary percutaneous coronary intervention (pPCI) due to STEMI is at major determinant of myocardial damage. In addition, increased thrombus burden increases the risk of periprocedural complications such as acute stent thrombosis and microvascular perfusion disorder due to distal embolization.^[3]^ Thrombosis triggered by inflammation is one of the cornerstones of STEMI pathophysiology.^[4]^ Recent studies have shown that increased C-reactive protein (CRP) and low serum albumin (SA) levels can be used as biomarkers of systemic inflammation, and are associated with adverse cardiovascular events.^[3]^ Many studies have shown that the CRP/SA ratio is more sensitive than these 2 parameters separately in predicting the systemic inflammatory status.^[5]^ To the best of our knowledge, no study has investigate the relationship between CAR and the coronary thrombus burden in newly diagnosed patients undergoing pPCI. This study investigated this relationship.

## 2. Materials and methods

### 2.1. Study population

Study retrospectively examined the data of 361 patients who visit our clinic between August 2022 and July 2023 due to newly diagnosed STEMI within the first 12 h of symptoms and underwent pPCI. STEMI was diagnosed based on the diagnostic criteria of the European Society of Cardiology guidelines.^[6]^ The exclusion criteria of the study were defined as more than 12 hours after the onset of symptoms (n = 12), cardiogenic shock (n = 2), ventricular tachycardia or ventricular fibrillation (n = 3), thrombolytic therapy applied in the last 24 hours, presence of active infection or autoimmune disease (n = 10), chronic liver failure (n = 3), receiving oral anticoagulant therapy (n = 8), active bleeding, severe kidney failure (estimated glomerular filtration rate less than 60 mL/min/1.73 m^2^ or dialysis) (n = 8), presence of prior coronary artery disease or PCI history (n = 68). In addition, 21 patients were excluded from the study because their information could not be fully accessed. Based on these criteria, 145 patients were excluded from the study, and the remaining 216 patients were included in the study. The study protocol was approved by the Ethics Committee of Bilecik Şeyh Edebali University Faculty of Medicine in accordance with the declaration of Helsinki and written informed consent was obtained from all participants.

### 2.2. Angiographic analysis

In this study, coronary angiograms stored in the hospital database were examined and all angiograms before intervention were evaluated for infarct-related arteries (IRA). The angiographic series of the IRA with the best image was chosen for thrombus analysis. The analysis of the angiograms was performed by 2 experienced cardiologists, blinded to the study design. The angiographic coronary thrombus grade was determined based on the thrombus burden seen after the total lesion was crossed with a guidewire or after percutaneous transluminal coronary angioplasty with a small balloon, according to the thrombolysis in myocardial infarction thrombus score. Accordingly, grade 0: no evidence of thrombus, grade 1: possible presence of thrombus, grade 2: largest thrombus size ≤ ½ vessel diameter, grade 3: twice the vessel diameter > linear thrombus size > ½ vessel diameter, grade 4: largest thrombus size ≥ twice the vessel diameter, grade 5 is defined as continued thrombotic total occlusion even after placement of a small balloon or guide wire. According to the thrombolysis in myocardial infarction score, patients with grades 1, 2 and 3 were classified into the low thrombus burden (LTB) group and those with grades 4 and 5 were classified into the high thrombus burden (HTB) group.^[7]^

### 2.3. Obtaining clinical and demographic data

Information related to cardiovascular risk factors such as diabetes mellitus, hypertension, smoking, family history of cardiovascular disease, and previously diagnosed cardiovascular disease, was obtained from the hospital record system. In addition, the medication us histories of all patients were recorded. Left ventricular ejection fraction (LVEF) values of the patients were obtained from echocardiographic recordings made within the first 24 hours following pPCI using the modified Simpson method. Routine biochemical and hemogram parameters were obtained from the analysis of blood samples taken from the antecubital region during hospital admission, to the hospital and conventional cardiac troponin T (cTnT) levels were recorded during hospitalization. SA and CRP levels were obtained from the results of blood samples taken immediately during admission to the emergency department. SA levels were measured with automatic photometry commercial kits using an Abbott C8000i (Abbott Park, IL), and CRP levels were measured using the nephelometric method (UniCel DxC 800 System; Beckman Coulter Inc, Brea, CA). CAR values were obtained by dividing the CRP level by the SA level.

### 2.4. Statistical analysis

Statistical analyses of the collected data were performed using Statistical Package for the Social Sciences (SPSS for Windows, version 22.0; IBM Corp., Armonk, NY). Continuous variables with a normal distribution a described as mean ± standard deviation and continuous variables without a normal distribution a described as median and interquartile range.

Categorical variables were expressed as percentages and compared using the chi-square or Fisher’s exact test, data normality was verified using the Kolmogorov–Smirnov test, and 2 groups were compared using the independent-samples *t* test for continuous data conforming to the normal distribution, non-normally distributed data were compared using the Mann–Whitney *U* test, the relationship between parameters was determined using Spearmen’s correlation coefficient, and receiver operating characteristic analysis was used to obtain the cutoff value of CAR for the prediction of HTB. The univariate and multivariate logistic regression analyses were used to identify independent predictors of HTB. Statistical significance was set at *P* < .05.

## 3. Results

**Figure 1.**
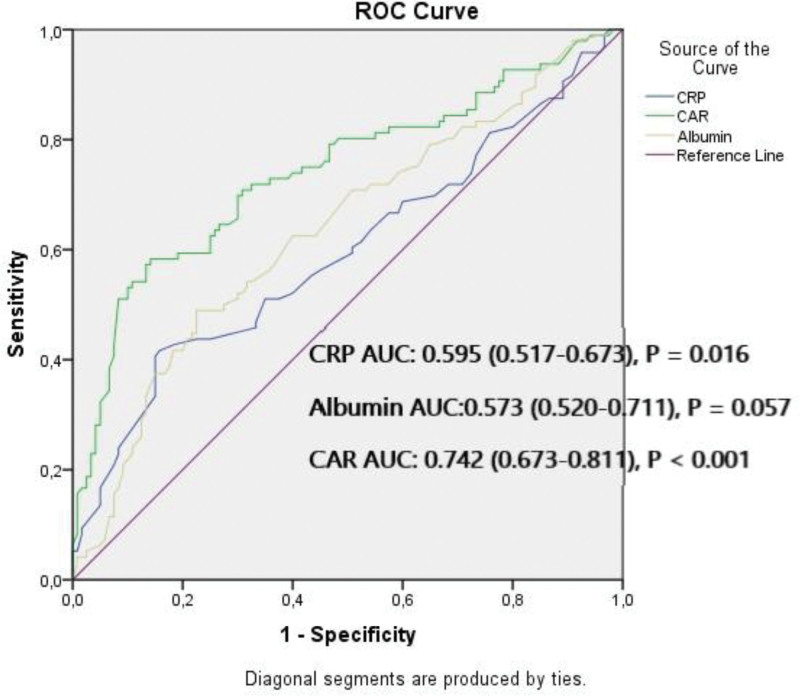
ROC curve analysis shows the predictive cutoff value of CAR for HTB. AUC = area under the curve, CAR = C-reactive protein to albumin ratio, CRP = C reactive protein, HTB = high thrombus burden, ROC = receiver operating characteristic curve.

A total of 216 newly diagnosed STEMI patients were included in the study, with 120 in the LTB group and 96 in the HTB group. The average age of the patients included in the study was 60 ± 9.8, and the male ratio was 61.1%. The baseline demographic, clinical, and laboratory results for both groups are shown in Table 1. Apart from age, other cardiovascular risk factors, medication usage history, IRA distribution, stent diameter and length, pain-balloon time, and lipid parameters were similar in both the groups (*P* > .05). Compared with the LTB group, the patients in the HTB group had higher age, SYNergy between PCI with TAXUS and Cardiac Surgery (SYNTAX) score, baseline cTnT, peak cTnT, CRP, CAR, glucose, white blood cell (WBC), and neutrophil/lymphocyte ratio values, while LVEF and albumin values were lower (*P* < .05). Spearman’s correlation analysis revealed a, moderate significant correlation between thrombus burden and CAR (*r* = 0.422, *P* < .001), age (*r* = 0.378, *P* < .001), SYNTAX score (*r* = 0.351, *P* = .001), baseline cTnT (*r* = 0.401, *P* < .001), neutrophil/lymphocyte ratio (*r* = 0.335, *P* < .001), glucose (*r* = 0.340, *P* < .001), and WBC count (*r* = 0.361, *P* < .001), and a weak significant relationship between thrombus burden and LVEF (*r* = −0.287, *P* < .001), CRP (*r* = 0.161, *P* < .001), and SA level (*r* = −0.257, *P* < .001) (Table 2). The highest correlation coefficient was found between CAR and thrombus burden. In the binary logistic analysis performed to determine independent predictor factors for HTB, it was revealed that SYNTAX score, CRP, CAR, glucose and WBC were independent and significant predictor factors for HTB (Table 3). According to the receiver operating characteristic analysis, when the cutoff value for CAR was ≥ 1.105, CAR could predict HTB with 70.8% sensitivity and 67.7% specificity (AUC: 0.742, confidence interval: 0.673–0.811, *P* < .001) (Fig. 1).

**Table 1:** Baseline demographic, clinical characteristics and laboratory parameters of study groups.

Variables	Low thrombus burden (n = 120)	High thrombus burden (n = 96)	*P*
Age (yr)	57 ± 9.3	63.8 ± 9	**<.001**
Male, n (%)	74 (61.7%)	58 (60.4%)	.851
Diabetes mellitus, n (%)	42 (35%)	38 (39.6%)	.488
Hypertension, n (%)	41 (34.2%)	45 (46.9%)	.058
Smokers, n (%)	37 (30.8%)	23 (24%)	.262
Hyperlipidemia, n (%)	46 (38.3%)	39 (40.6%)	.241
Family history of CAD, n (%)	35 (29.2%)	33 (34.4%)	.413
Medical history, n (%)			
Acetylsalic acid	35 (29.2%)	25 (26%)	.610
Statin	32 (26.7%)	20 (20.8%)	.319
Beta-blocker	37 (30.8%)	29 (30.2%)	.921
ACEI/ARB	32 (26.7%)	34 (35.4%)	.165
CCB	19 (15.8%)	15 (15.6%)	.902
İRA, n (%)			
LAD	45 (37.5%)	43 (44.8%)	.524
LCX	32 (26.7%)	21 (21.9%)	
RCA	43 (35.8%)	32 (33.3%)	
SYNTAX score	15.1 (10.3–18)	20.3 (15.6–25)	**<.001**
BMI (kg/m^2^)	27.7 (25–30.3)	26.9 (24.2–29.2)	.118
Stent diameter (mm)	3.09 ± 0.33	2.98 ± 0.27	.210
Stent length (mm)	22.8 ± 7.02	23.1 ± 6.71	.546
Pain to ballon time (min)	55.2 (45–60)	59.1 (40–78.8)	.564
LVEF (%)	44.9 (40–48)	41.3 (35.8–45)	**<.001**
Baseline cTnT (ng/mL)	285 (100–325)	596 (203–733)	**<.001**
Peak cTn T (ng/mL)	3225 (1890–4801)	5911 (4031–8412)	**<.001**
CRP (mg/dL)	3.8 (2.2–5.1)	4.5 (2.3–6)	**.016**
Albumin (d/L)	3.9 (3.8–4.2)	3.8 (3.5–4.1)	**.001**
CAR	0.93 (0.62–1.23)	1.33 (1.03–1.61)	**<.001**
Glucose (mg/dL)	137.5 ± 50.5	148.3 ± 54.1	**.023**
Creatinine (mg/dL)	0,91 ± 0.26	0.97 ± 0.25	.093
Hemoglobin (g/dL)	13.3 ± 1.41	12.8 ± 1.51	.101
WBC (×10^3^ µL)	11.2 ± 1.7	12.6 ± 2.1	**<.001**
NLR	1.76 (1.1–2.1)	2.85 (2.07–3.76)	**<.001**
Platelet (×10^3^ µL)	218 ± 65	212 ± 71	.233
Total cholesterol (mg/dL)	194.8 ± 32.3	198.1 ± 28.3	.445
Triglyceride (mg/dL)	183.4 ± 40	187.1 ± 45	.102
LDL-C (mg/dL)	142.5 ± 24.1	145 ± 21.8	.441
HDL-C (mg/dL)	35.6 ± 5.8	36.1 ± 6.4	.556

ACEI = angiotensin-converting enzyme inhibitor, ARB = angiotensin receptor blocker, BMI = body mass index, CAD = coronary artery disease, CAR = C-reactive protein to albumin ratio, CCB = calcium channel blockers, CK-MB = creatine kinase muscle brain, CRP = C-reactive protein, CTFC = correct thrombolysis in myocardial infarction frame count, CX = left circumflex artery, HDL-C = high-density lipoprotein cholesterol, IRA = İnfarct-related artery, LAD = Left anterior descending artery, LDL-C = low-density lipoprotein cholesterol, LVEF = left ventricular ejection fraction, MBG = angiographically myocardial blush grade, NLR = Neutrophil–lymphocyte ratio, RCA = right coronary artery, SYNTAX = SYNergy between percutaneous coronary interventin with TAXus, WBC = white blood cell.

*P* values in bold indicate clinical significance.

**Table 2:** Correlation between thrombus burden and clinical, laboratory parameters.

Variables	Thrombus burden	
	*r*	*P*
CAR	0.422	<.001
Albumin	−0.257	<.001
CRP	0.161	.018
Age	0.379	<.001
SYNTAX Score	0.415	<.001
LVEF	−0.287	<.001
Baseline cTnT	0.401	<.001
Glucose	0.340	<.001
WBC	0.361	<.001
NLR	0.335	.032

CAR = C-reactive protein to albumin ratio, CRP = C-reactive protein, LVEF = left ventricular ejection fraction, NLR = neutrophil–lymphocyte ratio, SYNTAX = SYNergy between percutaneous coronary interventin with TAXus, WBC = white blood cell.

**Table 3:** Effects of various variables on high thrombus burden in univariate and multivariate logistic regression analyses.

Variables	Univariate analysis		Multivariate analysis	
	OR (95% CI)	*P* value	OR (95% CI)	*P* value
Age	1.085 (1.049–1.121)	<.001	1.037 (0.995–1.082)	.084
SYNTAX score	1.143 (1.089–1.1200)	<.001	1.070 (1.002–1.141)	**.042**
LVEF	0.906 (0.864–0.951)	<.001	0.988 (0.923–1.057)	.717
Basline cTnT	1.002 (1.001–1.003)	<.001	1.001 (1.000–1.002)	**.027**
CRP	1.199 (1.042–1.379)	.011	0.727 (0.552–0.958)	**.023**
Albumin	0.255 (0.115–0.567)	.001	0.384 (0.126–1.173)	.093
CAR	7.660 (3.745–15.669)	<.001	10.206 (2.987–34.872)	**<.001**
Glucose	1.011 (1.004–1.017)	.001	1.009 (1.000–1.018)	**.042**
WBC	1.476 (1.257–1.734)	<.001	1.252 (1.041–1.505)	**.017**
NLR	2.466 (1.832–3.318)	<.001	1.374 (0.964–1.957)	.079

CAR = C-reactive protein to albumin ratio, CI = confidence interval, CRP = C-reactive protein, LVEF = left ventricular ejection fraction, NLR = neutrophil–lymphocyte ratio, OR = odds ratio, SYNTAX = SYNergy between percutaneous coronary interventin with TAXus, WBC = white blood cell.

*P* values in bold indicate clinical significance.

## 4. Discussion

To the best of our knowledge, this study is the first to investigate the relationship between CAR and thrombus burden in patients undergoing pPCI due to newly diagnosed STEMI. Despite the potent antiplatelet and anticoagulant treatment options developed today, the frequency of massive coronary thrombus during pPCI is around 16.4%, and thrombus burden is a risk factor for distal microembolization, no-reflow, and long-term adverse cardiovascular events.^[8–10]^ In this study, CAR, obtained using inexpensive and easily accessible laboratory parameters, was an independent factor for intracoronary thrombus burden in patients undergoing pPCI due to STEMI.

CAR is a marker related to the inflammatory and nutritional status, including CRP and SA parameters. Previous studies have shown that CAR is a marker for both prognosis and progression in patients with cancer.^[11]^ Recent studies support the use of CAR as a marker of cardiovascular diseases and have shown that CAR is associated with the no-reflow phenomenon and acute stent thrombosis.^[12,13]^ Rupture of atherosclerotic plaques and subsequent total occlusion of the coronary artery constitutes the fundamental mechanism of STEMI. Increased intracoronary thrombus burden in acute coronary syndrome is associated with no-reflow and distal embolization, and increased intracoronary thrombus burden causes deterioration in the left ventricle, which functions as a perfusion disorder at the microvascular level despite successful pPCI performed in STEMI patients.^[14]^ Indeed, similar to these findings, LVEF was found to be lower in HTB patients in our study, and cTnT level, which is an indicator of myocardial injury area, was found to be higher in patients with HTB. During STEMI, proinflammatory substrates released from necrotic myocardial cells cause an inflammatory response both locally and systemically; CRP is a well-known acute phase reactant and is used as a marker for atherosclerosis.^[15]^ Previous studies have shown that CRP localizes in the infarct area, combines with complement factors in this region, and is stored. It is also thought that the complement fixed on the cell surface triggers the coagulation cascade.^[16]^ Additionally, CRP is believed to play a role in endothelial damage and activation of lymphocytes and platelets accumulated in this region.^[17]^ In our study, the CRP levels were higher in the HTB group. SA, another component of CAR, is the major determinant of plasma oncotic pressure and also has a protective anti-inflammatory effect as an acute phase reactant. It is thought that SA also has a protective effect against endothelial damage and thrombotic state caused by free oxygen radicals, cytokines such as interlokin-6 and tumor necrosis factor alpha, and these protective mechanisms are insufficient in hypoalbuminemia. It is also thought that SA, which is the major component of plasma viscosity, has a negative correlation with erythrocyte aggregation and inhibits platelet activation and aggregation. In addition to all these, it is known that the synthesis of lipid and coagulation factors increases in hypoalbuminemia, and then a hypercoagulable state occurs. SA, an other component of CAR, inhibits platelet aggregation by directly and indirectly increasing PGD2 production.^[18–20]^ Indeed, in this study, SA levels were lower in the HTB group. In this study, in parallel with the mechanisms above, it was shown that CAR is an independent factor for HTB. It was also shown that CAR was more effective than CRP and albumin separately in predicting HTB.

In our study, it was shown that the initial troponin level is an independent risk factor for HTB. This, which is consistent with the findings of Pawłowski et al.^[21]^ The authors revealed the presence of a relationship between initial troponin and thrombus burden determined by optical coherence tomography in patients undergoing pPCI due to acute coronary syndrome. Another important result of our study is that the SYNTAX score was shown to be a risk for HTB. Recent studies have shown the relationship between the severity of coronary artery disease determined by the SYNTAX score and impaired endothelial dysfunction. It is believed that endothelial dysfunction, which develops due to impaired nitric oxide release in patients with a high SYNTAX score, causes HTB.^[22]^

Another striking result of this study is that hyperglycemia has been shown to be an independent risk factor for HTB. Theories related to the relationship between hyperglycemia and thrombus burden are contradictory; however, 2 fundamental mechanisms are proposed: first, the contribution of the hemodynamic and hormonal changes, as a result of large infarct size caused by thrombus burden, to increase blood glucose concentrations; and second, the increased thrombogenic condition due to platelet dysfunction caused by hyperglycemia itself.^[23]^

Another aspect of this study that warrants discussion is that it showed that WBC is also a risk factor for HTB. This, which is in line with the findings of Barron et al.^[24]^ The inflammatory response that develops after plaque rupture leads to thrombogenic activation and the materials released after plaque rupture further enhance platelet activation.^[25]^ In our study, a significant correlation between the SYNTAX score and thrombus burden was demonstrated, and the SYNTAX score was found to be higher in patients with HTB. Therefore, we believe that the increased prevalence of coronary artery disease leads to an increased inflammatory response, resulting in enhanced thrombogenic activation.

## 5. Limitations

The retrospective nature of the study and the small number of patients included in the study are an important limitation. Another limitation could be the evaluation of thrombus burden based on angiographic assessment, which is influenced by many factors, instead of a more objective method such as optical coherence tomography.

## 6. Conclusion

This study revealed that CAR is an independent and significant factor for coronary thrombus burden in patients admitted to pPCI due to STEMI. It was also demonstrated that the efficacy of CAR alone in predicting thrombus burden is greater than using CRP and SA levels separately. We believe that predicting coronary thrombus burden, which is closely related to mortality, with this test obtained from inexpensive and easily accessible laboratory parameters could guide treatments in this patient group. However, we believe that larger-scale studies are needed to prove the effectiveness of this parameter for use in clinical practice.

## Author contributions

**Conceptualization:** Mustafa Kaplangoray, Kenan Toprak, İsa Ardahanli.

**Data curation:** Mustafa Kaplangoray, Kenan Toprak, Ramazan Aslan, Edhem Deveci, Ahmet Gunes, İsa Ardahanli.

**Formal analysis:** Mustafa Kaplangoray, Ramazan Aslan, Edhem Deveci, Ahmet Gunes.

**Investigation:** Mustafa Kaplangoray, Kenan Toprak, Ramazan Aslan, Edhem Deveci, Ahmet Gunes.

**Methodology:** Mustafa Kaplangoray.

**Project administration:** Mustafa Kaplangoray.

**Resources:** Mustafa Kaplangoray, Kenan Toprak, Ramazan Aslan, Edhem Deveci, Ahmet Gunes, İsa Ardahanli.

**Software:** Mustafa Kaplangoray.

**Supervision:** Mustafa Kaplangoray, İsa Ardahanli.

**Validation:** Mustafa Kaplangoray, İsa Ardahanli.

**Visualization:** Mustafa Kaplangoray.

**Writing – original draft:** Mustafa Kaplangoray, Kenan Toprak.

**Writing – review & editing:** Mustafa Kaplangoray, Kenan Toprak, İsa Ardahanli.
